# Can supplements with prebiotic fibres positively influence bone health in type 2 diabetes? Insights from a randomised controlled crossover trial

**DOI:** 10.1007/s11657-025-01556-x

**Published:** 2025-06-05

**Authors:** Eline Birkeland, Wuraola Aduke Bamigbetan, Kristine Duus Molven, Per M. Thorsby, Hanne L. Gulseth, Anne-Marie Aas, Cecilie Dahl

**Affiliations:** 1 Section of Nutrition and Dietetics, Division of Medicine, Department of Clinical Service, Oslo University Hospital, 0424 Oslo, Norway; 2 Section for Clinical Data Management, Research Support for Clinical Trials, Oslo University Hospital, Oslo, Norway; 3 Department of Nutrition, Institute of Basic Medical Sciences, University of Oslo, Oslo, Norway; 4 Hormone Laboratory, Department of Medical Biochemistry and Biochemical Endocrinology and Metabolism Research Group, Oslo University Hospital, Aker, Oslo Norway; 5 Division of Mental and Physical Health, Norwegian Institute of Public Health, 0213 Oslo, Norway; 6 Institute of Clinical Medicine, University of Oslo, 0316 Oslo, Norway; 7 Institute of Health and Society, University of Oslo, 0318 Oslo, Norway

**Keywords:** Bone turnover markers, Calcium, Magnesium, Nutrient uptake, Prebiotics, Type 2 diabetes, Vitamin D

## Abstract

**Summary:**

Inulin-type fructans did not significantly improve serum levels of calcium, magnesium, vitamin D, or bone turnover markers in people with type 2 diabetes (T2D). However, interactions between gut microbiota and bone health were suggested, indicating the need for further research in this population.

**Background:**

Evidence suggests that a healthy gut microbiome benefits bone health, especially in immunocompromised populations like the elderly and people with T2D.

**Objective:**

We investigated the effect of prebiotics (inulin-type fructans) on serum concentrations of calcium, magnesium, 25(OH) vitamin D, and the bone turnover markers N-terminal propeptide of type 1 collagen (P1NP), and C-terminal telopeptide of type 1 collagen (CTX-1) in people with T2D.

**Design:**

Participants (29) were treated for 6 weeks with 16 g inulin-type fructans and 16 g control supplement (maltodextrin) in randomised and double-blind crossover design, with a 4-week washout between treatments.

**Results:**

Compared to the control, inulin-type fructans did not significantly affect serum concentrations (mean ± SEM) of calcium (0.05 ± 0.02 mmol/L vs. 0.02 ± 0.03 mmol/L, p = 0.324), magnesium (0.02 ± 0.01 mmol/L vs. 0.00 ± 0.01 mmol/L, p = 0.352), 25(OH) vitamin D (-3.60 ± 1.94 nmol/L vs. -2.00 ± 1.97 nmol/L, P = 0.564), P1NP (0.81 ± 0.95 ug/L vs. -0.89 ± 0.97 ug/L, p = 0.210), or CTX-1 (-0.01 ± 0.01 ug/L vs. 0.00 ± 0.01 ug/L, p = 0.438). However, post hoc analyses of correlations between changes support that cross-talk between the human host and gut microbiota may influence bone health in this population.

**Conclusion:**

This study does not support that inulin-type fructans may improve serum levels of calcium, magnesium, or 25(OH) vitamin D, nor that they affect bone turnover markers in people with T2D over 6 weeks. Interactions between microbiota and bone health in this population warrants further investigations.

The trial is registered at clinicaltrials.gov (NCT02569684).

**Supplementary Information:**

The online version contains supplementary material available at 10.1007/s11657-025-01556-x.

## Introduction

Bones store essential minerals such as calcium and magnesium, which contribute to bone rigidity and are released into the body as needed to maintain mineral balance [1]. Vitamin D is crucial for calcium absorption, while magnesium activates vitamin D, which helps regulate calcium metabolism. Bone remodelling is a tightly coordinated process of bone formation and resorption that ensures calcium levels remain within a critical range [2]. This process can be monitored using sensitive biomarkers such as the N-terminal propeptide of type I collagen (P1 NP) and the C-terminal telopeptide of type I collagen (CTX-1). P1NP is cleaved from procollagen during bone matrix formation, while CTX-1 is released when cross-linked collagen in bone breaks down [3]. In most cases, both markers increase with high bone turnover and decrease with low bone turnover. In conditions involving an overall increase in bone turnover, such as osteoporosis, P1NP and CTX-1 are typically elevated [4].

Osteoporosis is a condition that has a significant health impact on an aging society because of its high morbidity, mortality, and healthcare costs [5]. The risk of developing osteoporosis increases with age and affects both men and women, although women are particularly vulnerable to this condition after menopause.

People with type 2 diabetes (T2D) face an increased risk of developing osteoporosis [6]. Despite often having a higher body mass index (BMI), which seems to protect against osteoporosis in a healthy population [7] and maintain high or normal bone mineral density (BMD), their risk of fractures remains elevated [8]. The exact mechanism underlying bone impairment in T2D remains unclear. One key theory suggests that excessive adipocyte formation from stem cells and expansion of adipose bone marrow tissue may occur at the expense of osteoblast differentiation and bone formation [9, 10]. Accompanied by elevated osteoclast activity driven by pro-inflammatory cytokines, this may negate the protective effect of high BMI on osteoporosis in T2D [9, 10]. Diabetes duration, type of medication, glycaemic control, and diabetes complications may further influence osteoporosis risk [8, 9].

In recent years, evidence has emerged demonstrating interactions between gut bacteria and bone homeostasis [11]. The human host and gut microbiome have a mutualistic relationship, where the microbial genome produces enzymes not coded for in the human genome. These enzymes are necessary to break down prebiotic fibres including inulin-type fructans (ITF) and galacto-oligosaccharides (GOS) [12]. Substrates selectively utilized by host microorganisms conferring a health benefit have been defined as prebiotics [13, 14]. Several animal studies report beneficial effects of dietary fibres with prebiotic quality on general bone health and intestinal absorption of calcium and magnesium [11, 15, 16]. Studies in healthy postmenopausal women and adolescence have also shown improved calcium and magnesium status after treatment with prebiotic fibres [11, 15]. The microbial fermentation of these indigestible fibres leads to the production of short-chain fatty acids (SCFA) such as butyric, acetic and propionic acid. SCFA have been suggested to act as signalling molecules affecting bone turnover (osteoclast activity) [17]. Furthermore, the bacterial metabolism of prebiotic fibres into SCFA lowers intestinal pH, which in turn may facilitate the absorption of minerals such as calcium and magnesium [12, 17, 18]. Another possible mechanism is the influence of gut bacteria on bone homeostasis via the immune system by inhibiting the release of pro-inflammatory cytokines such as TNF-α, IL-6, and IL-1β that play a role in osteoclast formation [17].

A limited amount of studies have investigated the effect of prebiotics on bone markers in humans, and a few appear to have found improvement, but not significant changes [19–28].

To our knowledge, only one study has explored and found a positive influence of prebiotics on calcium status in people with T2D [21], and the present study may be the first to also investigate the effects of ITF on magnesium status and bone turnover markers in this population. The aim of this study was thus to investigate the effect of ITF on serum concentrations of calcium, magnesium, 25(OH) vitamin D and bone turnover markers (CTX-1/P1NP) in people with T2D.

As a secondary objective, we wanted to explore associations between the concentrations and changes in gut microbiota (bacterial species) and SCFA on changes in serum concentrations of calcium, magnesium, 25(OH) vitamin D, and bone turnover markers.

## Methods

This randomised, double-blind, and placebo-controlled crossover trial was conducted at the Diabetes Research Laboratory of Oslo University Hospital, Aker from February 2016 to December 2017. We previously reported moderate alterations in the composition of gut microbiota and increased faecal concentrations of SCFA after treatment with ITF in T2D and the effects on hormones, glycaemic regulation or appetite [29–31]. The present paper reports secondary outcomes from the study.

### Participants

The study included adult men and women diagnosed with T2D and a BMI of 40 kg/m^2^ or less. Participants were not on insulin or GLP-1 analogue medication and had an HbA1c level below 10.0% (86 mmol/mol). Exclusion criteria comprised pregnancy, recent antibiotic treatment within the last three months, recent weight fluctuations exceeding 3 kg within the past month, use of prebiotic or probiotic supplements, dietary fibre intake over 30 g/day, substance abuse, or engagement in high-intensity exercise. Individuals with chronic conditions that could influence study outcomes or hinder participation, such as inflammatory bowel disease, were also excluded. Fibre intake was evaluated using a simplified method, asking participants about the frequency and portion sizes of specific high-fibre foods commonly consumed in Norway.

### Sample size

Calculation of sample size related to the outcome of the main study investigating the effect of prebiotics on GLP-1 and glycaemic control [32]. Few previous studies with relevant data were available for estimation of sample size. Thus, the calculation of sample size was based on changes in area under the curves (AUCs) for GLP-1 response in people with T2D in a pharmaceutical trial [33]. Accordingly, a sample size of 23 individuals was needed to achieve 80% power at α of 0.05.

However, in a crossover study conducted by Holloway et al*.* [25] with 15 postmenopausal women, the prebiotic treatment group exhibited an average standard deviation of 10.7% difference in magnesium absorption between the baseline and 6-week measurements. Accordingly, with 25 participants included in the present study, we had 80% power to detect an 8.8% or larger difference in magnesium absorption at 6 weeks compared to baseline, using a significance level of 0.05.

### Study recruitment

Participants were recruited via social media platforms, the Diabetes Outpatient Clinic at Oslo University Hospital, informational posters in the hospital lobby and local pharmacies, as well as through general practice referrals. Eligibility was assessed during a screening visit conducted at least four weeks prior to enrolment.

**Fig. 1:**
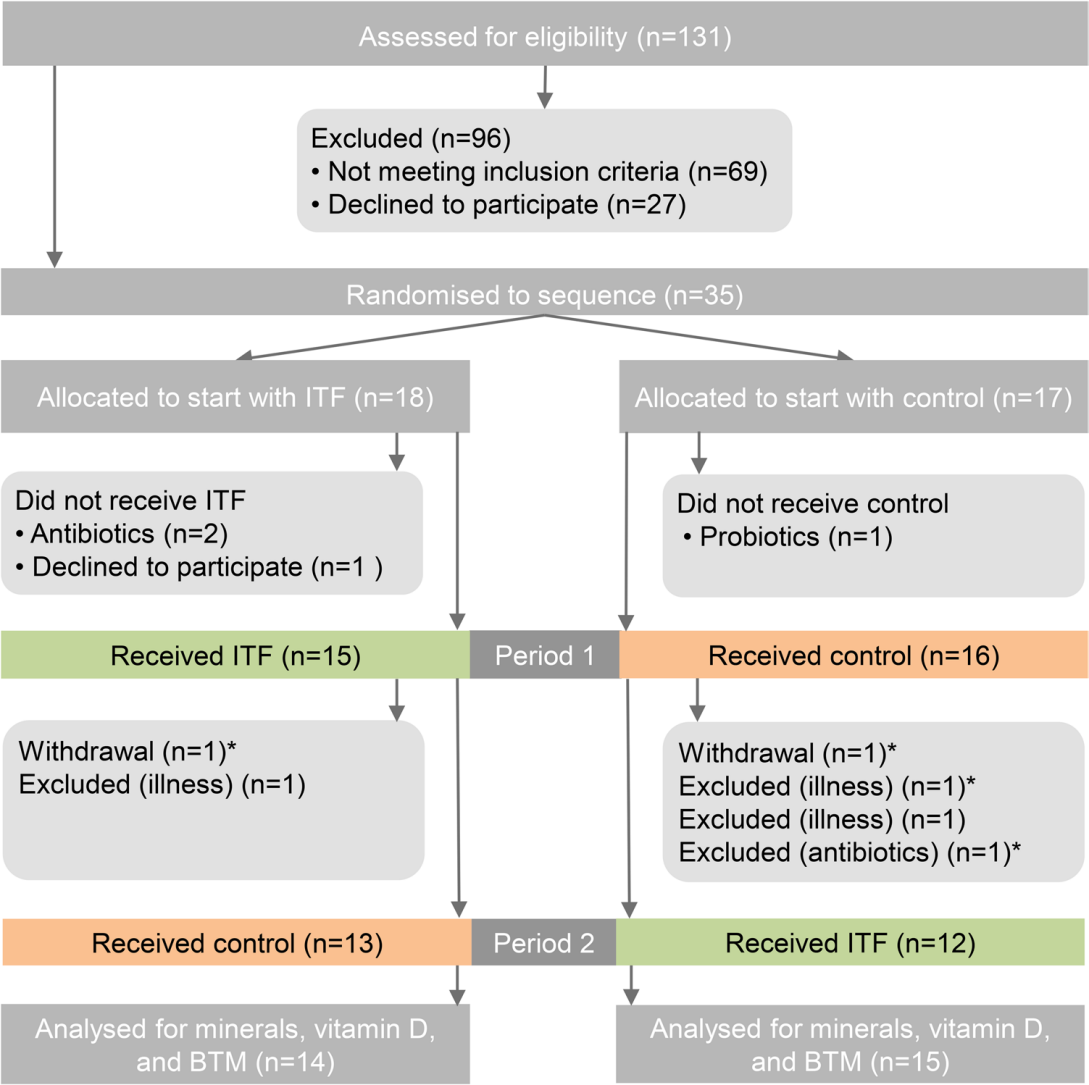
Diagram illustrating study flow and all subjects screened for eligibility. *Included in the analyses; ITF, inulin-type fructans; BTM, bone turnover markers

We evaluated 131 subjects, ultimately assigning 35 to begin treatment with either ITF or a control. (Fig. 1). The primary reason for exclusion was long distance from the participants' homes to the study centre. Before the study commenced, four participants withdrew, and an additional six participants dropped out during the course of the study. Three participants voluntarily withdrew, citing personal reasons. Three others were excluded due to the initiation of antibiotic treatment for minor infections. One participant was excluded for using a probiotic supplement, and three participants were diagnosed with serious illnesses, rendering them unable to continue. Twenty-nine participants were included in the analysis for calcium, magnesium, 25(OH) vitamin D, CTX and P1NP. Twenty-five participants attended all four visits. The participants were asked to keep up their usual routines throughout the study. They were instructed to discontinue diabetes medication two days before each visit, avoid intense physical activity one day prior, and begin fasting from midnight before the visits. Blood samples were collected early in the morning.

### Intervention and data collection

**Fig. 2:**
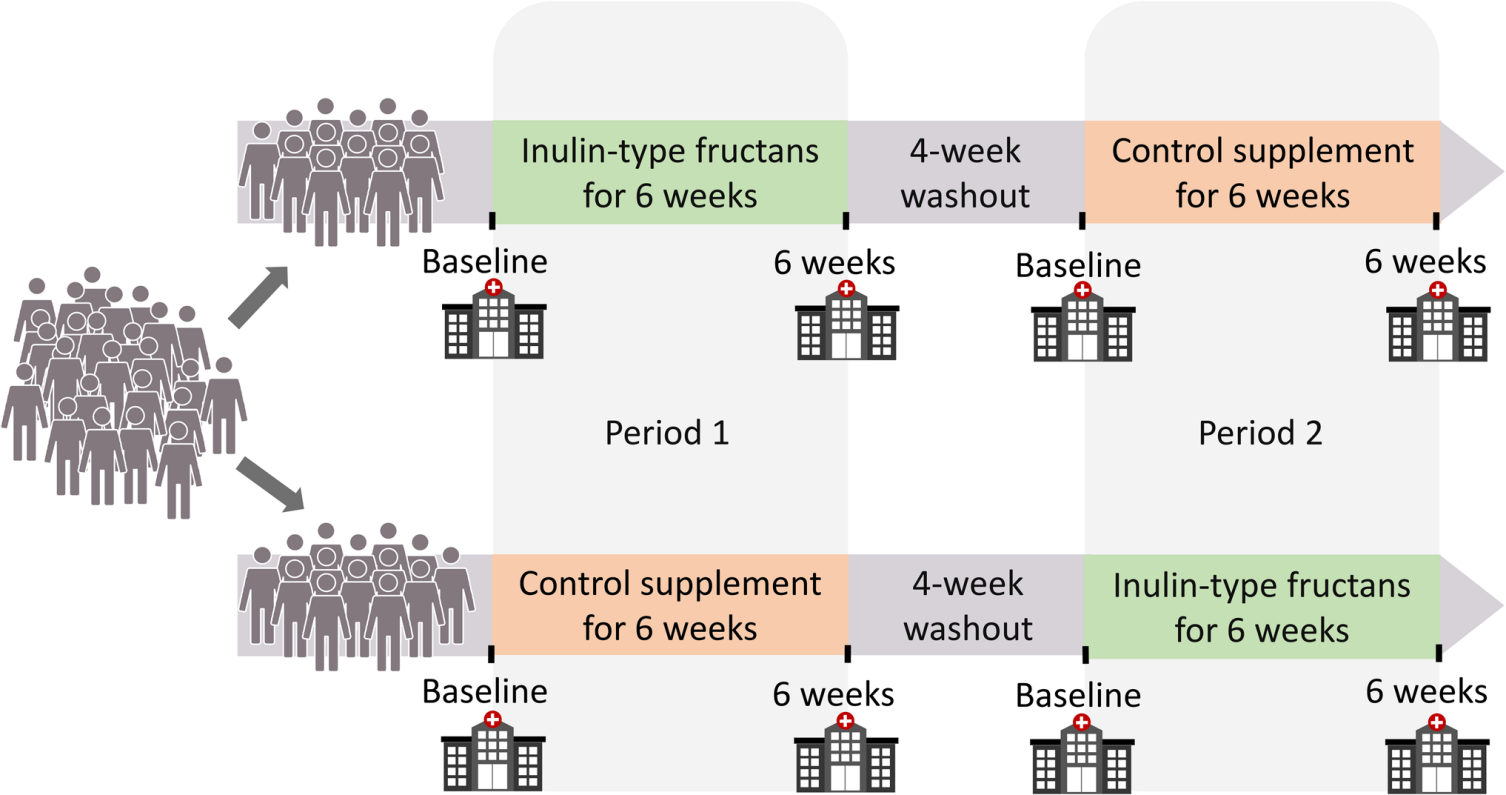
Overview of the study design. Abbreviation: ITF, inulin-type fructans

The participants underwent a 6-week treatment with 16 g Orafti® Synergy1 (Beneo GmbH, Germany; a 50/50 mixture oligofructose and inulin) and 16 g maltodextrin as control (Agrana Stärke, Austria; AGENAMALT 20.222 Maltodextrin DE19) in a randomised, double-blind crossover design (Fig. 2). A 4-week washout period was included between the treatments. The supplements were provided in powdered form, indistinguishable in flavour and appearance, and packaged in uniform, opaque 8-g sachets. Participants blended the powders with meals or beverages and consumed them at their convenience. The participants were directed to consume one sachet each day during the first week to allow for gastrointestinal adaptation, and then increase to two sachets per day the proceeding five weeks.

To supplement the existing data on gut microbiota and SCFA, we analysed already collected fasting serum samples stored at − 80 °C in biobank, to determine the concentration of bone-essential nutrients (calcium, magnesium and vitamin D) and bone turnover markers (P1NP and CTX-1). Calcium and magnesium in serum were analysed using a photometric method on equipment from Roche Diagnostics, CV 4,5% and 3% respectively, at the Biochemistry Laboratory, Oslo University Hospital, Norway. 25(OH) vitamin D was analysed with liquid chromatography-mass spectrometry (LC–MS), coefficient of variation (CV) was 10–17%, and P1NP and CTX-1 were analysed with electrochemiluminescence immunoassay (ECLIA), CV 9% and 6% respectively, at the Hormone Laboratory, Oslo University Hospital, Norway. All methods were accredited according to NS-EN ISO 15189:2012.

The participants filled in a self-administered food frequency questionnaire (FFQ) at baseline about their eating habits regarding the latest six weeks [34] and a master’s student in the research group reviewed the forms with them to clarify any misunderstandings or suspicions of errors.

### Randomisation

Computer randomisation ensured that the allocation process was free from any unintentional bias by the researchers. Both participants and research staff were blinded to treatment allocation, and the statistician who conducted the computer randomisation and the research nurse administering supplements had no other involvement in the study. The randomisation key remained sealed until all data was gathered, the database cleaned, and the laboratory analyses completed.

### Statistical analyses

SPSS version 29.0 software was used for descriptive statistics. Biochemical responses were analysed using Stata for Windows (Version 18.0, Stata Corporation, College Station, TX, USA). Baseline characteristics are summarised as mean (SD) or n (%). We inspected potential relations between baseline (first baseline) data with Pearson correlation, or Spearman rank correlation if appropriate. Linear mixed model (LMM) was used to compare responses between and within interventions. Repeated measures were regarded in accordance with best model fit. Fixed effects in the LMM models were treatment (prebiotics/control) and day (baseline/6 weeks), with their interactions. In all models, the effect of period and treatment order was considered and kept if significant. We investigated the influence of sex, age, baseline BMI, diabetes duration, and metformin as confounding factors. Microbiota and faecal SCFA were significantly affected by the prebiotic treatment, as previously reported [29], and baseline (first baseline) levels of micronutrients determined from FFQs were also explored in the models. Normality of residuals was evaluated with quantile–quantile (QQ)-plots and Shapiro–Wilk test, and the outcome measures transformed if appropriate. Results were reported as LMM model-based means with 95% confidence intervals. Reported *p*-values were two-sided, and *p* < 0.05 was considered significant for all tests. *Post hoc* analyses with repeated measures correlation (rmcorr) were used to quantify the correlation between changes in the outcome measures without taking the intervention into account.

### Ethics

The study was approved by the Regional Ethics Committee for Medical and Health Research and registered at clinicaltrials.gov (NCT02569684), and all participants gave their written informed consent.

## Results

Twenty-nine participants were included in the statistical analyses (Fig. 1). Baseline characteristics are presented in Table 1. As a group, the participants reported unexpectedly high fibre intake but appeared otherwise representative of Norwegian patients with T2D. Supplemental figures S1-S7 show correlation between the serum values of P1NP and CTX-1, dietary intake of vitamin D and serum P1NP, serum values of vitamin D and P1NP, BMI and serum CTX-1, serum magnesium and age, and serum values of calcium and vitamin D in women.

**Table 1:** Subject characteristics at baseline^a^

Variable		*n* = 29
Women		12 (41%)
Age (years)		61.5 ± 11.7
Diabetes duration (years)		5.1 ± 4.4
BMI (kg/m^2^)		28.9 ± 4.5
Diastolic blood pressure (mmHg)		136.3 ± 17.9
Systolic blood pressure (mmHg)		85.6 ± 9.5
HbA_1C_ (% [mmol/mol])		6.9 ± 1.0 [52]
Serum calcium (mmol/L)		2.30 ± 0.10
Serum magnesium (mmol/L)		0.83 ± 0.07
Serum 25(OH) vitamin D (nmol/L)		65.0 ± 20.1
Serum P1NP (µg/l)		38.6 ± 12.1
Serum CTX-1 (µg/l)		0.32 ± 0.15
Dietary fibre (g/day)		31.5 ± 10.2
Dietary calcium (mg)		1091 ± 478
Dietary magnesium (mg)		490 ± 173
Dietary vitamin D (µg)		18.1 ± 11.1
Diabetes treatment		
	Diet	8 (27.6)
	Metformin	21 (72.4)
	SLGT2 inhibitors	4 (13.8)
	DPP-4 inhibitors	7 (24.1)
	Sulfonylureas	1 (3.4)

*BMI* body mass index, *HbA*_*1c*_ glycated haemoglobin, *P1NP* procollagen type I N-propeptide, *CTX-1* C-terminal telopeptide of type 1 collagen, *SLGT2* sodium glucose cotransporter-2, *DPP4* dipeptidyl peptidase-4. ^a^ Data are mean ± SD or *n* (%)

### Effect of treatments on outcomes

**Fig. 3:**
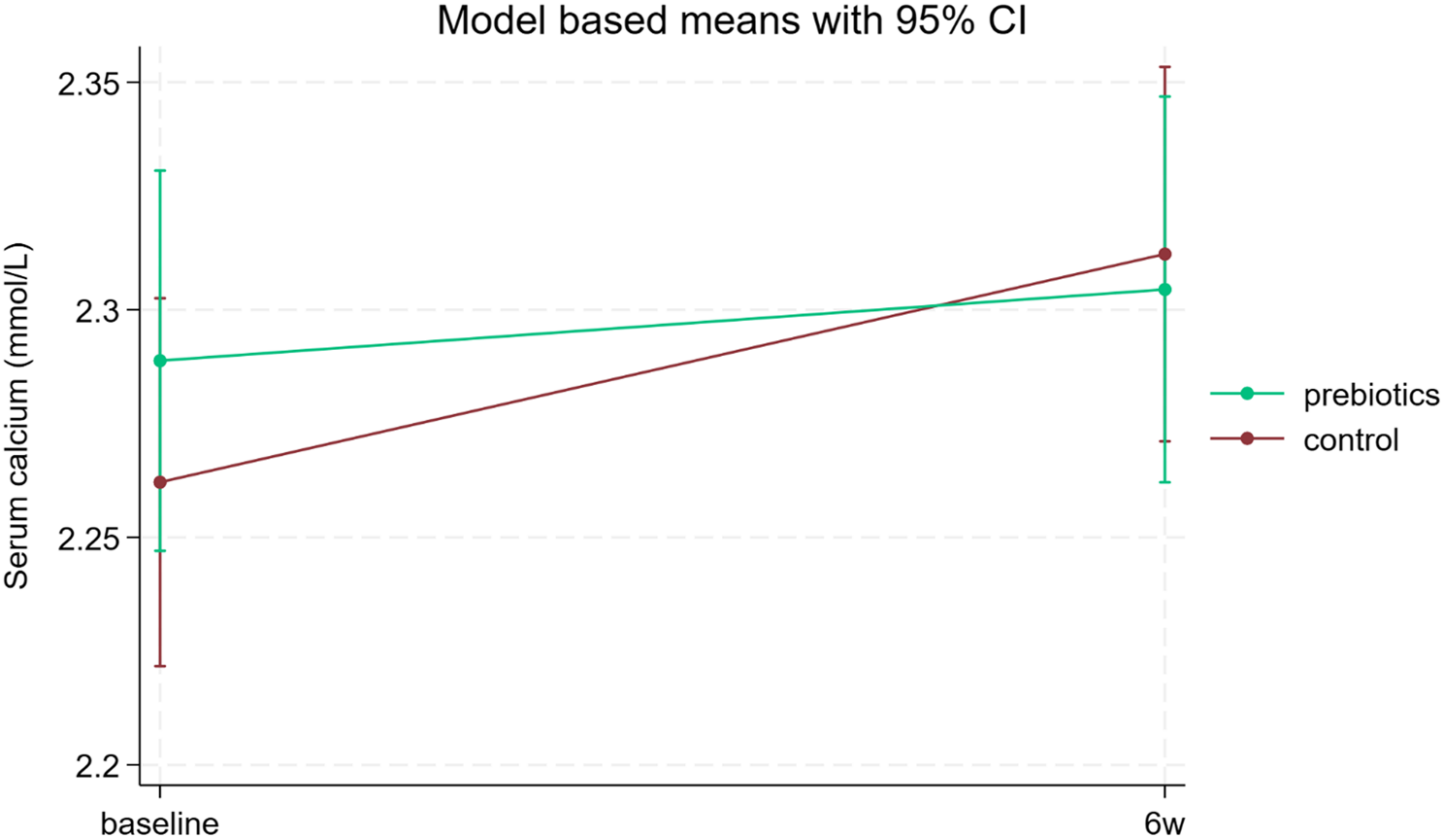
Serum calcium responses before (baseline) and after (6 weeks) treatment with prebiotics and a control supplement, adjusted for period, sex and serum 25(OH) vitamin D

**Fig. 4:**
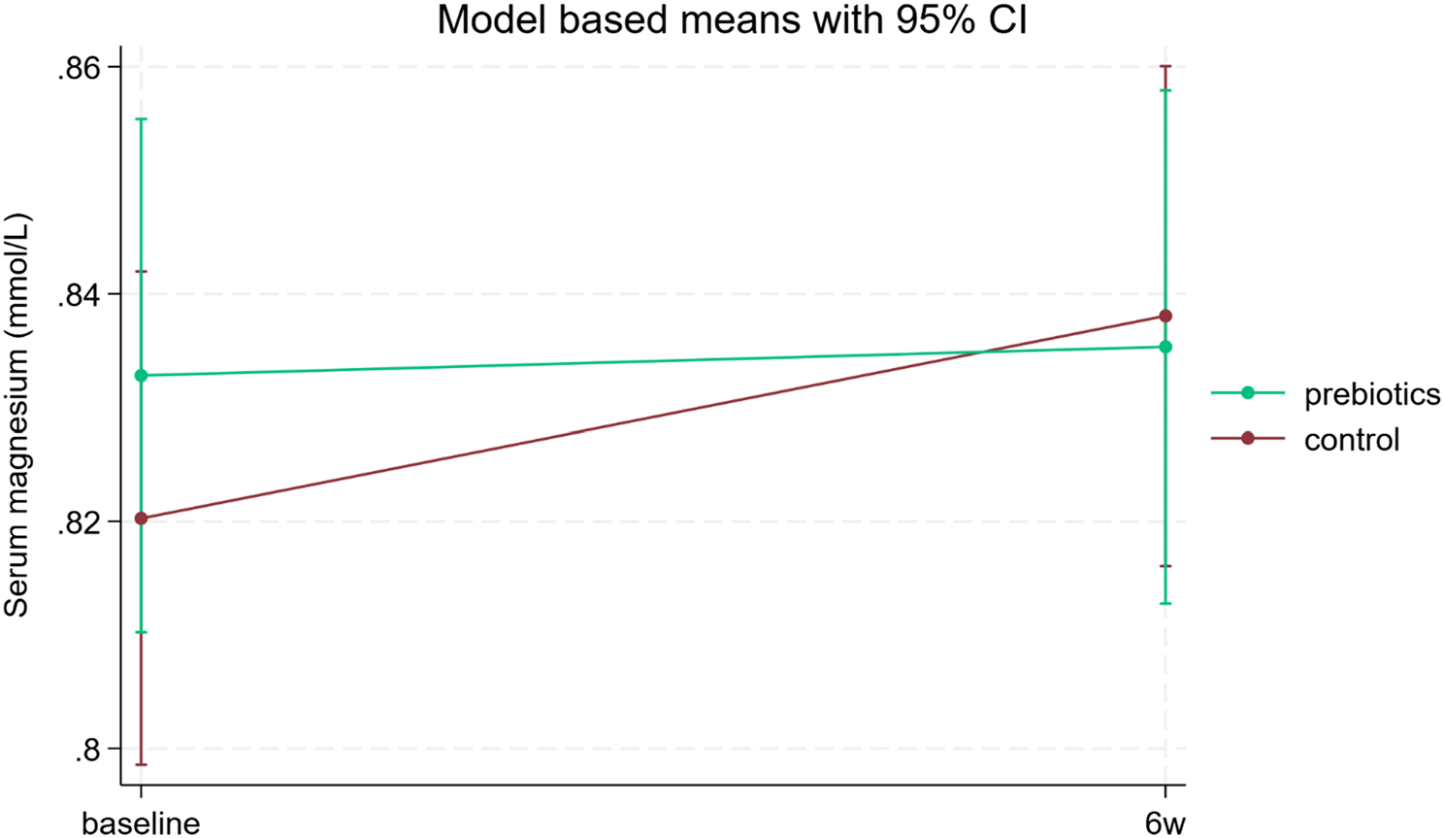
Serum magnesium responses before (baseline) and after (6 weeks) treatment with prebiotics and a control supplement

Results from the LMM are shown in Table 2 and Fig. 3, 4, S8, S9, and S10. We found no significant positive effects of the prebiotic supplement on any of the outcomes. The analysis of serum calcium showed significant effects of period, sex and serum vitamin D. There was also a significant positive effect of the control supplement on serum calcium that was not significantly different from the effect of prebiotics. (Table 2). Neither microbiota nor faecal SCFA significantly influenced the estimates. The randomisation order had no influence on any of the outcomes, and there were no significant differences between the two baseline values (before and after washout) for any of the outcomes.

**Table 2:** Model-based means and 95% confidence intervals of calcium, magnesium, 25(OH) vitamin D and BTMs in serum during treatment with ITF and control supplement^a^

	Prebiotics					Control supplement					
	baseline	95% CI	6 weeks	95% CI	Within -*p*	baseline	95% CI	6 weeks	95% CI	Within -*p*	Between *-p*
Calcium^b^ (mmol/L)	2.29	2.25–2.33	2.31	2.26–2.35	0.534	2.26	2.21–2.30	2.31	2.27–2.25	0.040	0.324
Magnesium (mmol/L)	0.83	0.81–0.86	0.84	0.81–0.86	0.832	0.82	0.80–0.85	0.84	0.81–0.86	0.151	0.401
25(OH) vitamin D (nmol/L)	64.9	57.8–72.1	62.7	55.5–69.9	0.265	63.4	56.3–70.6	59.9	52.8–67.1	0.071	0.643
P1NP (μg/L)	37.3	32.9–41.7	36.5	32.1–40.9	0.395	39.0	34.6–43.4	39.8	35.4–44.2	0.396	0.230
CTX-1 (μg/L)	0.31	0.26–0.36	0.31	0.26–0.36	0.432	0.32	0.27–0.37	0.31	0.26–0.36	0.775	0.451

*BTM* bone turnover marker, *CI* confidence interval, *P1NP* procollagen type I N-propeptide, *CTX-1* C-terminal telopeptide of type 1 collagen.^a^ Data are model-based means and 95% CI; ^b^ Adjusted for period, sex and serum 25(OH) vitamin D

### *Post hoc* analyses

When exploring changes in the outcomes without considering the interventions, we found some modest correlations. Changes in P1NP correlated inversely to changes in the genus *Bifidobacterium*, r_m_ (95% CI) = −0.25 (−0.45,−0.02), *p* = 0.030, and to *Bifidobacterium adolecentis*; r_m_ (95% CI) = −0.26 (−0.46,−0.04), *p* = 0.022. An inverse relation between changes in serum calcium and changes in the Lachnospiraceae family was seen, but did not reach significance; r_m_ (95% CI) = −0.21 (−0.42, 0.02) *p* = 0.066. Changes in serum vitamin D related positively to faecal acetic acid; r_m_ (95% CI) = 0.26 (−0.04, 0.46), *p* = 0.0220 and negatively to changes in faecal butyric acid; r_m_ (95% CI) = −0.31 (−0.50,−0.09), *p* = 0.006. Serum CTX-1 changed negatively with faecal acetic acid, r_m_ (95% CI) = −0.22 (−0.42,−0.01), p = 0.055.

## Discussion

In the current investigation, six weeks of treatment with 16 g of ITF did not significantly affect serum concentrations of calcium, magnesium, 25(OH) vitamin D, or the bone turnover markers P1NP and CTX-1 in people with T2D. However, modest correlations were found between changes in species from the genus *Bifidobacterium* and changes in the bone marker P1NP, and between changes in the SCFAs acetic and butyric acid and changes in serum vitamin D in *post hoc* analyses, suggesting that gut bacteria and their metabolites indeed may play a role in the rate of bone turnover in T2D.

We investigated the association between ITF intake and *serum levels* of calcium, magnesium, vitamin D, and change in BTMs. Serum levels of nutrients such as calcium are tightly regulated through bone resorption and kidney excretion [35]. However, they can still provide insight into the body's homeostatic balance, which may serve as an indirect indicator of bone health. Few studies have investigated the effect of prebiotics on serum calcium levels [35]. Even so, far more studies have explored the treatment potential of ITF on the absorption of calcium (a key factor in overall calcium metabolism) and bone turnover, and some have analysed the effect of ITF on magnesium absorption and bone strength [19–28, 36–43].

GOS and other dietary fibres with prebiotic qualities, such as resistant starch and soluble corn fibre, have also been studied. The studies vary widely in populations, design, methodology, and present conflicting results. Among studies that reported a positive effect of ITF on calcium absorption, the majority had either considerably larger sample size, higher treatment dose or longer treatment duration than the present study [21, 25, 36–40]. A study conducted by Holloway et al*.* found that 10 g of ITF per day for 6 weeks, improved calcium (and magnesium) absorption in 15 postmenopausal women in a crossover study [25]. However, several studies found no effect of ITF on calcium absorption [20, 23, 24, 26, 27, 41–43]. Among these, one study stands out due to its particularly large sample size and extended treatment duration: Reyes-Garcia et al*.* conducted a 3-arm parallel study involving 461 postmenopausal women, administering 5 g of ITF daily for 2 years. It could be argued that a 5 g dose per day may have been insufficient in adults, as the lowest dose used in studies reporting positive effects on calcium absorption was 10 g per day in adults [21, 25, 37], and 8 g per day in children [39, 40].

Some studies reported improved calcium absorption in adolescent children and postmenopausal women after treatment with soluble corn fibre or GOS [18, 44, 45]. Two studies found no change in calcium absorption in postmenopausal women with a history of bariatric surgery or men with or without hyperinsulinemia after treatment with soluble corn fibre or resistant starch [46, 47].

Studies showing a positive effect of ITF on magnesium absorption all had equal or shorter treatment durations, smaller sample sizes, and lower treatment doses compared to the present study [24, 25, 48]. Other studies, however, found no significant impact on magnesium absorption [36, 41, 42].

Among the many studies exploring changes in bone turnover markers following ITF treatment, none showed significant effects when compared to placebo [19–28]. However, it should be noted that most of these studies were small, and the lack of findings may be attributed to type-II errors. In our exploratory analyses, we observed that variations in *Bifidobacterium* species and SCFAs may impact BTMs and vitamin D levels. This study is among the first to investigate these relations. However, a previous study on patients who underwent laparoscopic sleeve gastrectomy and experienced gut microbial changes reported similar findings, specifically noting higher PN1P levels with reduced microbial diversity and increased CTX levels with lower faecal butyrate [49].

We did not assess bone mass or BMD, and only a few other studies have. To see an effect on BMD, a longer treatment period is needed, and there may also be variations in effects by age. Abrams et al*.* conducted a year-long study involving 98 adolescent boys and girls, who were daily administered 8 g of ITF or a placebo, both mixed in calcium-fortified juice [39]. The ITF treatment significantly improved total bone mineral content and BMD compared to the placebo. However, in postmenopausal women, two studies reported no effect of daily ITF intake on BMD over a year, possibly due to the lower doses used (3.6 g and 5 g) [20, 22]. As oestrogen deficiency is known to reduce calcium absorption, the effect of prebiotics on BMD may also be influenced by participants’ hormonal status and physiologic state at baseline [22, 50]. The absorption of calcium with supplements of prebiotics has previously been found to be higher when oestrogen levels were low (i.e. as in the early follicular phase periods of the menstrual cycle and in the postmenopausal stage) and in participants with a lower initial spinal BMD and osteopenia [22, 25, 50].

### Strengths and limitations

As previously reported, compliance was high and the participants reported somewhat more passage of gas and flatulence after treatment with ITF (16 participants) compared to control treatment (2 participants) (P < 0.001) [30]. There were no significant changes in other gastrointestinal symptoms or any adverse effects during the trial.

With expectations of high inter-individual response of gut microbiota to dietary influence [51], the crossover design was a strength in this study, enabling the participants to be their own controls. Regrettably, we did not obtain menopausal status in the female participants. Menopausal status could have had an influence on the effect of ITF due to variations in oestrogen level. However, only 2 of the 12 participating women were under 52 years old (the median age for menopause).

While P1NP and CTX are the preferred BTMs according to the International Osteoporosis Foundation, they are still subject to analytical variability influenced by factors such as circadian rhythms, pre-sample food intake, and sample handling. Additionally, individual differences, including renal function, may further impact results. In the current study, serum samples were collected in fasting state at the same time each day, and the participants were compared to themselves, reducing the variability in measurements. The samples were also handled in one laboratory using the same standardized procedure. Still, BTMs are intended as adjunct assessment tools in osteoporosis management alongside DXA scanning and is, therefore, not a direct measure of bone health.

The FFQ employed in this study is validated for evaluating habitual diet in an adult Norwegian population and designed to capture dietary intake over the past year [34]. However, our participants were instructed to recall their diet from the last six weeks when completing the questionnaires as the primary purpose of the FFQ was to ensure that no significant dietary changes occurred during the study period. This adjustment is unlikely to have compromised the FFQ’s purpose in this trial and may even have reduced recall bias, a common issue with retrospective dietary assessment methods.

Results from the FFQ indicated higher intake of dietary fibre at baseline than anticipated. Due to the participants’ knowledge of the nature of the study, the possibility of over reporting cannot be ruled out. Healey et al*.* found more pronounced bifidogenic response from prebiotic treatment when habitual fibre intake was high [52], but as previously reported we did not make the same observation in our study sample [29].

In retrospect, it could be speculated whether a larger sample size or longer treatment duration would have been more appropriate. Yet, studies with smaller sample sizes [24, 25, 36, 48] and shorter treatment durations [24, 36, 38, 40, 48] found significant effects of ITF over placebo on absorption of calcium and magnesium. Few studies have investigated whether or not prebiotic intake have an effect on *serum levels* of calcium and magnesium. In addition, none of these were able to demonstrate an effect on bone turnover markers, regardless of sample size or duration. Nevertheless, the study’s small sample size and short duration of the intervention in the present study may have weakened the study power, preventing us from determining whether the treatment impacted bone health. The daily dose of ITF (16 g) was intentionally limited to mitigate gastrointestinal side effects. Although compliance appeared excellent, this was only crudely measured by the number of returned and unused sachets.

One of the many challenges in clinical research involving microbiota is the multitude of influencing factors, with the list of recognised elements expanding rapidly. There may thus be unknown factors attributed to populations with T2D, which render treatment with ITF ineffective to bone health.

## Conclusions

The current investigation did not find a significant improvement in the serum levels of magnesium, calcium, vitamin D, or bone turnover markers after treatment with prebiotics for 6 weeks among people with T2D. However, the *post hoc* analyses of correlations between changes in SCFA and outcome measures, as well as changes between gut bacteria and outcome measures suggest that interactions between the human hosts and gut microbiota may indeed influence bone health in people with T2D. More investigations in participants with T2D will assist for better data comparability and data validation of the benefit of prebiotics on bone health in this population.

## Supplementary Information

Below is the link to the electronic supplementary material. 

ESM1 (DOCX 6.59 MB)

## Data Availability

Data supporting the findings of this study are available from the corresponding author upon reasonable request.
