# Supplementary material for: Can supplements with prebiotic fibres positively influence bone health in type 2 diabetes? Insights from a randomised controlled crossover trial
Source: Arch Osteoporos. 2025 Jun 5;20(1):71. doi: 10.1007/s11657-025-01556-x (PMC12141403; doi:10.1007/s11657-025-01556-x)
Supplement: Supplementary file 1 — (DOCX 6.59 MB) [file 11657_2025_1556_MOESM1_ESM.docx]

Supporting information

# Can supplements with prebiotic fibres positively influence bone health in type 2 diabetes? Insights from a randomised controlled crossover trial

Eline Birkeland^1^, Wuraola Aduke Bamigbetan^2^, Kristine Duus Molven^3^, Per M. Thorsby^4,6^, Hanne L. Gulseth^5^, Anne-Marie Aas^1,6,^ Cecilie Dahl^7^

^1^Section of Nutrition and Dietetics, Division of Medicine, Department of Clinical Service, Oslo University Hospital, 0424 Oslo, Norway

^2^Section for Clinical Data Management, Research support for Clinical Trials, Oslo University Hospital, Norway

^3^Department of Nutrition, Institute of Basic Medical Sciences, University of Oslo

^4^Hormone Laboratory, Department of Medical Biochemistry and Biochemical endocrinology and metabolism research group, Oslo University Hospital, Aker, Oslo, Norway

^5^Division of mental and physical health, Norwegian Institute of Public Health, 0213 Oslo, Norway

^6^Institute of Clinical Medicine, University of Oslo, 0316 Oslo, Norway

^7^Institute of Health and Society, University of Oslo, 0318 Oslo, Norway

Anne-Marie Aas and Cecilie Dahl share last authorship.

Corresponding Author: Cecilie Dahl, cecilie.dahl@medisin.uio, Department of Public Health Science, Institute of Health and Society, University of Oslo, Address: Postboks 1130 Blindern, 0318 Oslo, Norway

Serum P1NP and serum CTX-1 were positively correlated at baseline (*p*<0.001) (Fig. S1), Dietary intake of vitamin D correlated with P1NP at baseline (*p*=0.042) (Fig. S2), but not with CTX-1 (*p*=0.836) (figure not shown). Serum vitamin D was correlated with P1NP at baseline (*p*=0.038) (Fig. S3), but not with CTX-1 (*p*=0.329) (figure not shown). Subjects with higher BMI had lower CXT-1 at baseline (*p*=0.030) (Fig. S4). Serum magnesium correlated positively with age at baseline (*p*=0.035) (Fig. S5). Women had 4.0% higher concentrations of serum calcium compared to men at baseline (*p*=0.009) (Fig. S6), and serum calcium correlated positively with serum vitamin D only for the women (Fig. S7).


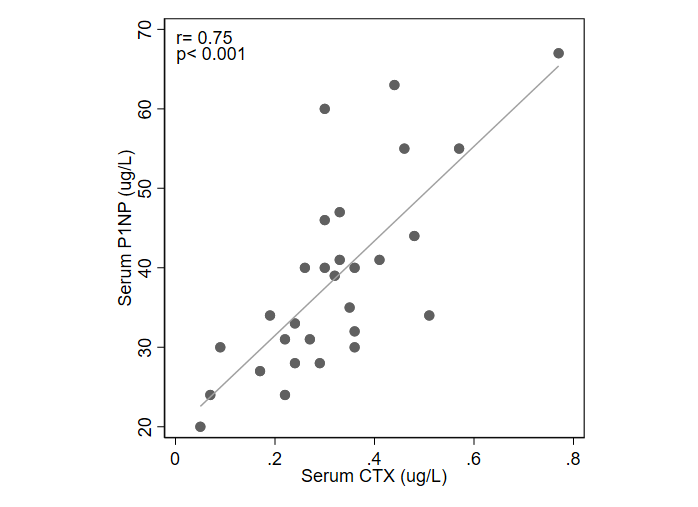


**Supplemental fig. 1** Pearson correlation between serum CTX and serum P1NP at baseline. Abbreviations: CTX, C-terminal telopeptide of type 1 collagen; P1NP, procollagen type I N-propeptide


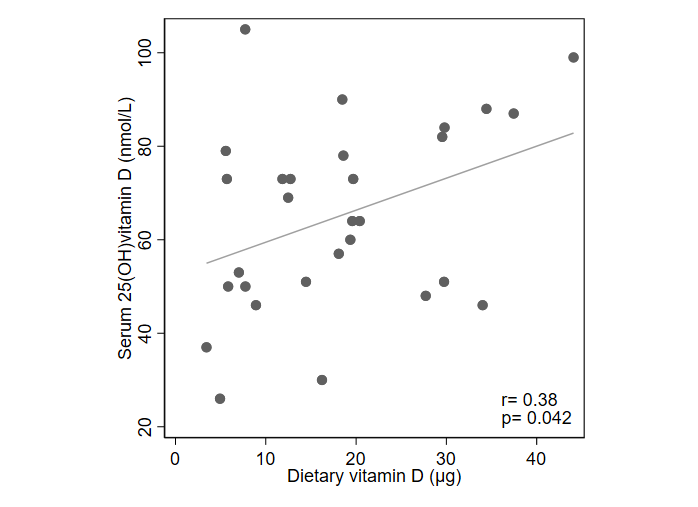


**Supplemental fig. 2** Pearson correlation between dietary vitamin D and serum 25(OH) vitamin D at baseline


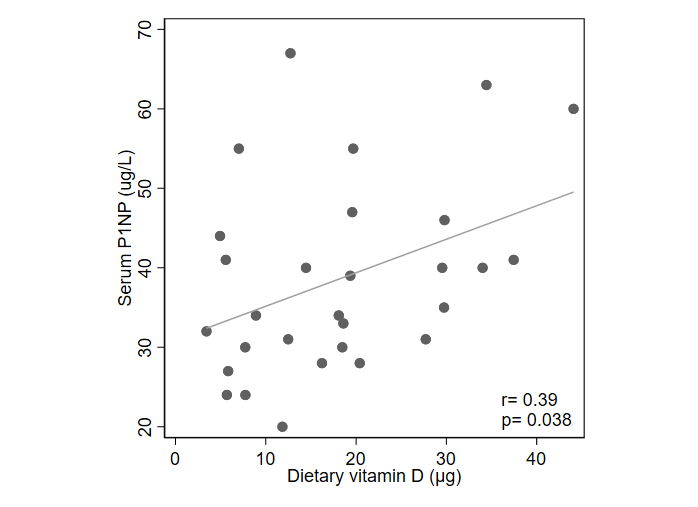


**Supplemental fig. 3** Pearson correlation between dietary vitamin D and serum P1NP at baseline. Abbreviation: P1NP, procollagen type I N-propeptide


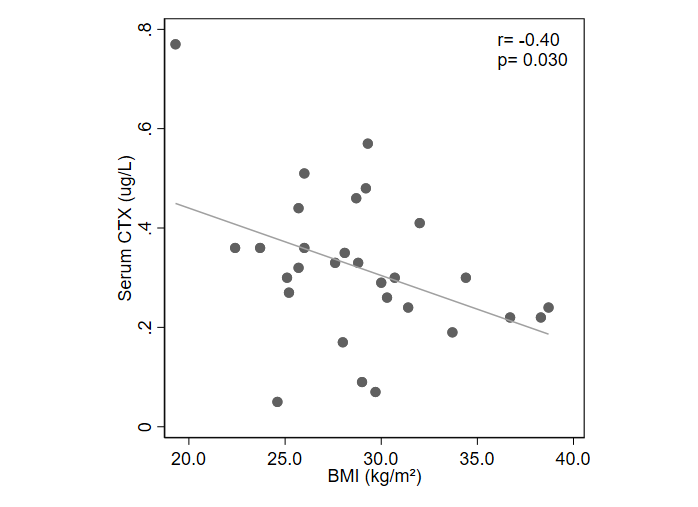


**Supplemental fig. 4** Pearson correlation between BMI and serum CTX at baseline. Abbreviations: BMI, body mass index; CTX, C-terminal telopeptide of type 1 collagen


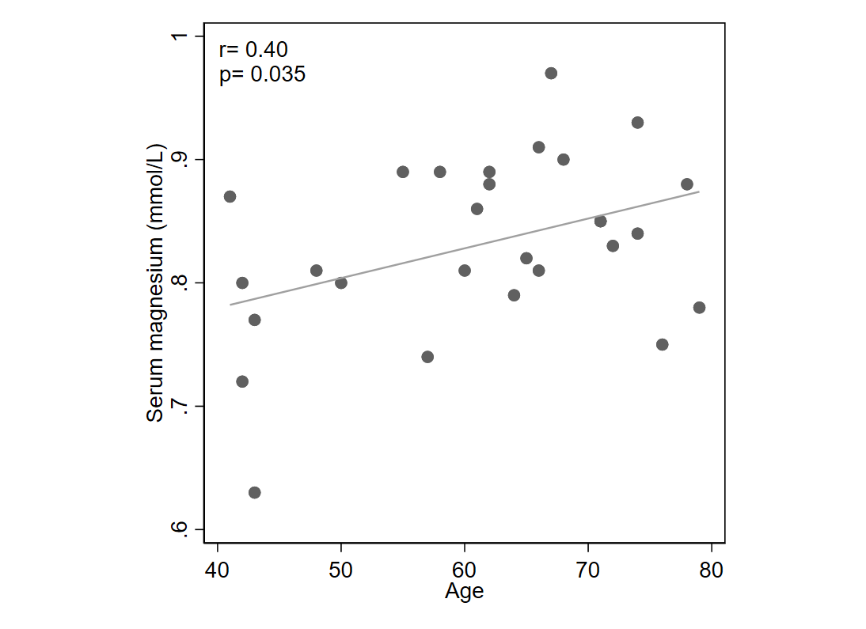


**Supplemental fig. 5** Pearson correlation between age and serum magnesium at baseline


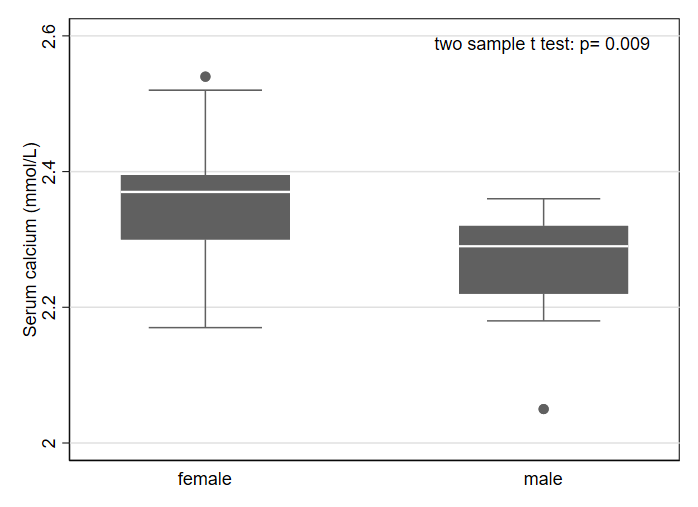


**Supplemental fig. 6** Box plot showing difference between women and men in serum calcium concentrations at baseline. Student’s independent-sample t test


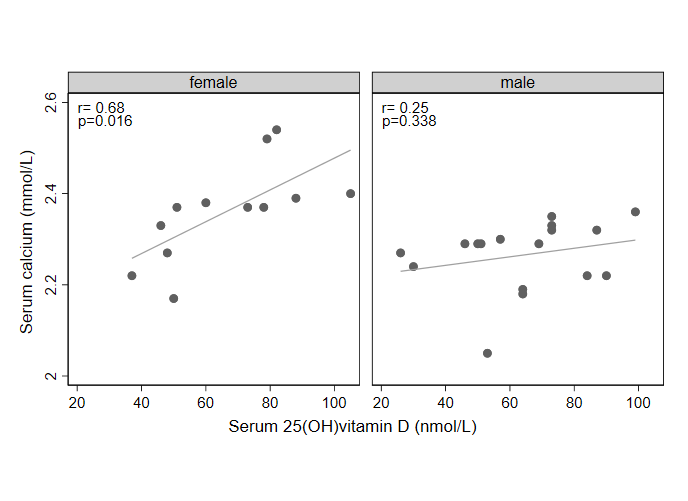


**Supplemental fig. 7** Pearson correlation between serum calcium and serum 25(OH) vitamin D at baseline shown separately for women and men


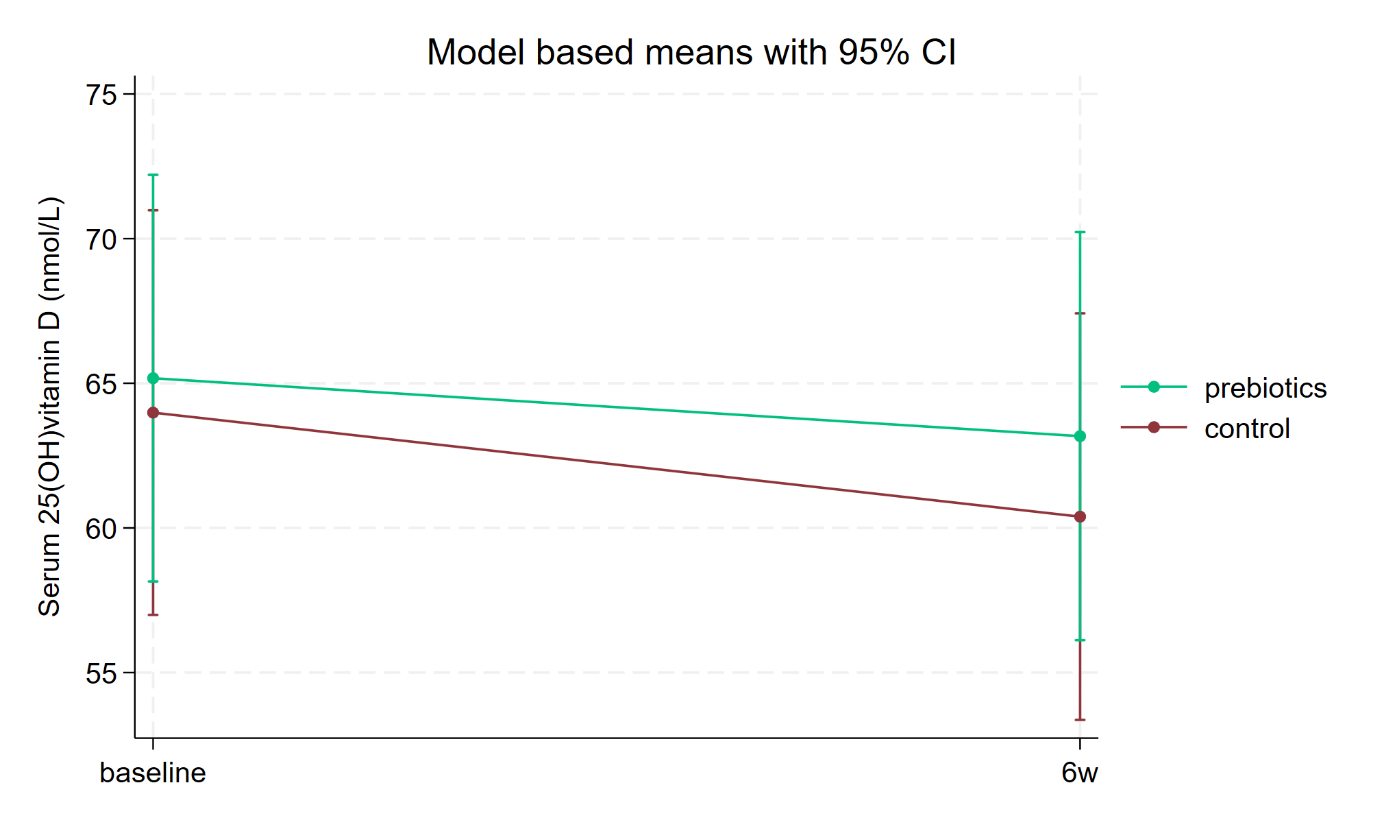


**Supplemental fig. 8** Serum 25(OH) vitamin D responses before (baseline) and after (6 weeks) treatment with prebiotics and a control supplement


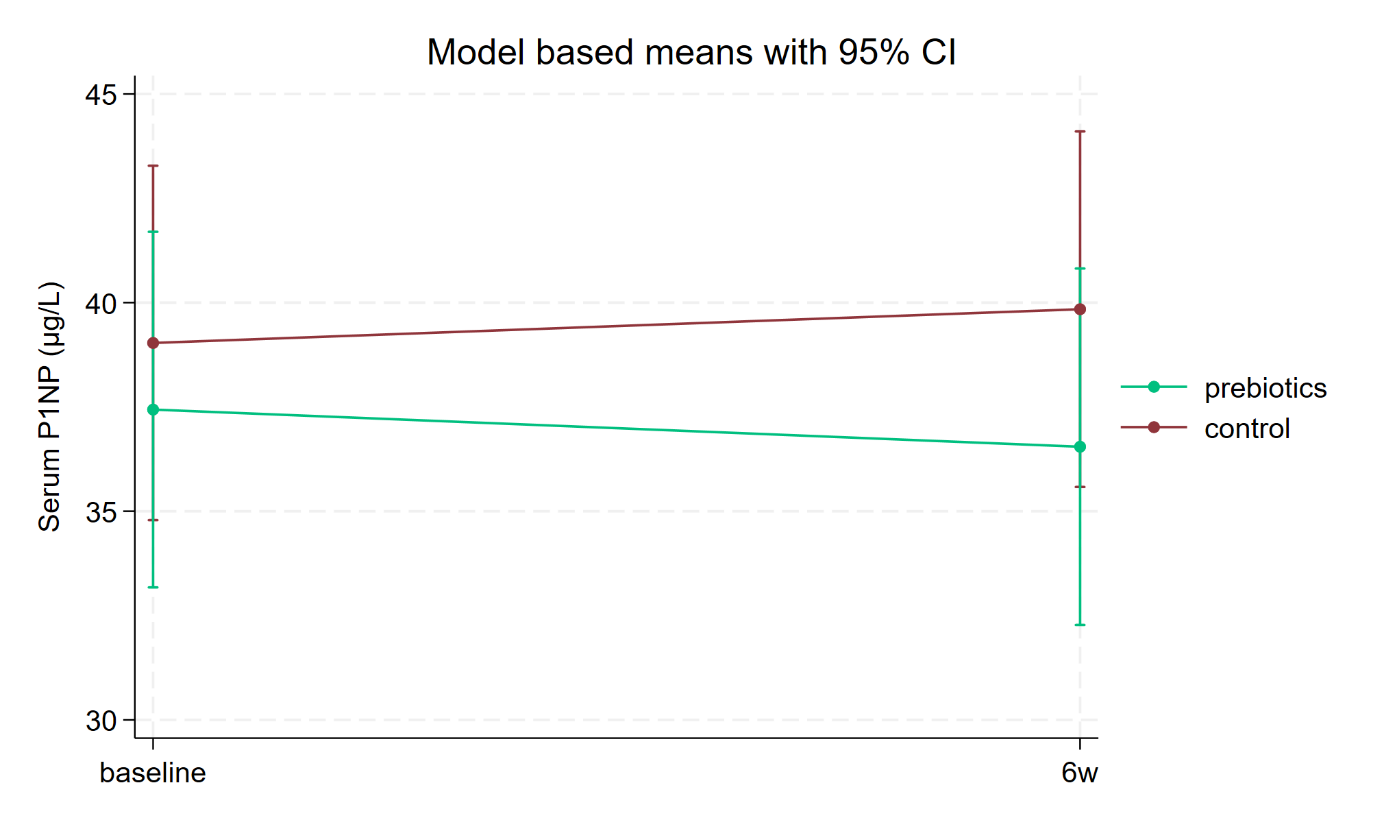


**Supplemental fig. 9** Serum P1NP responses before (baseline) and after (6 weeks) treatment with prebiotics and a control supplement. Abbreviation: P1NP, procollagen type I N-propeptide


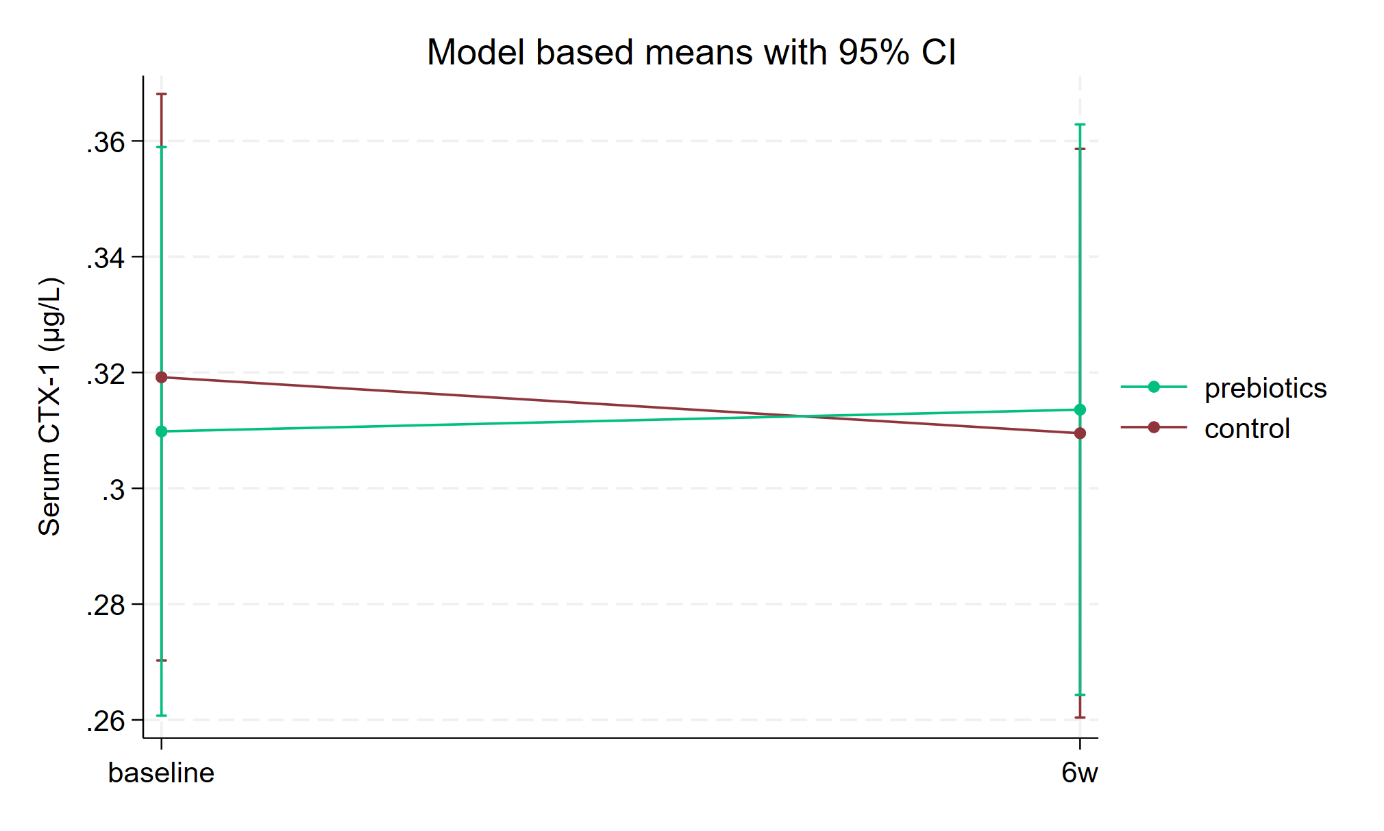


**Supplemental fig. 10** Serum CTX-1 responses before (baseline) and after (6 weeks) treatment with prebiotics and a control supplement. Abbreviation: CTX-1, C-terminal telopeptide of type 1 collagen
